# Anthropometric and Physical Fitness Profiles of World-Class Male Padel Players

**DOI:** 10.3390/ijerph17020508

**Published:** 2020-01-13

**Authors:** Cristóbal Sánchez-Muñoz, José Joaquín Muros, Jerónimo Cañas, Javier Courel-Ibáñez, Bernardino Javier Sánchez-Alcaraz, Mikel Zabala

**Affiliations:** 1 Department of Physical Education and Sport, University of Granada, 52071 Melilla, Spain; csm@ugr.es; 2 Department of Didactics of Musical, Plastic and Corporal Expression, University of Granada, 18071 Granada, Spain; 3 Department of Physical Education and Sport, University of Granada, 18071 Granada, Spain; jerocanias@yahoo.com (J.C.); mikelz@ugr.es (M.Z.); 4 Department of Physical Education and Sport, University of Murcia, 30720 Murcia, Spain; javier.courel.ibanez@gmail.com (J.C.-I.); bjavier.sanchez@um.es (B.J.S.-A.)

**Keywords:** body composition, somatotype, anthropometry, physical fitness, padel

## Abstract

The aims of this study were to describe and compare the anthropometric and physical fitness attributes of male padel players according to their competitive level, and to establish a functional anthropometric and physical profile. A total of 60 males participated in the present study. Athletes were grouped according to competition level, forming an elite group (n = 25) and a subelite group (n = 35). Anthropometric variables, hand grip and lumbar isometric strength, flexibility, and lower-body muscular strength were measured. Elite padel players were significantly older and showed significantly lower values for the thigh and calf skinfolds, the sum of six and eight skinfolds, and the sum of lower-limb skinfolds than the subelite group. Elite padel players also had significantly lower values than subelite players for body fat percentage and thigh fat area, whilst showing significantly higher values for lumbar isometric strength. Somatotype of the elite padel players could be defined as endo-mesomorphic. Results suggest that training and talent identification of padel players should focus on their anthropometric characteristics and physical fitness, with these being different between elite and subelite athletes. Normative data could help coaches throughout the talent identification process and in the design of training programs when seeking to optimise sports performance.

## 1. Introduction

Padel is a modern racket sport that was invented in the 1970s. With interest growing in recent years, padel has now become one of the most popular sports in Spain with over four million regular participants and it is rapidly spreading around the world [1]. In padel, two pairs of players confront each other inside an enclosed synthetic glass and metal court (20 × 10 meters). An important peculiarity is the existence of walls and grilles surrounding the court off of which the ball can bounce. This consequently lengthens rallies so the number of actions and strokes per player is higher in comparison with similar racket sports like badminton, tennis, or squash [2]. Previous studies examining its game dynamics and match activity have defined padel as a high-intensity intermittent activity, which combines high-frequency (0.7–1.5 per second (s)) and low-intensity actions during rallies that are of a moderate duration (9–15 s), interspersed by 1020 s of rest in between, leading to longer breaks of 90 s [3,4,5,6,7]. Offensive success in padel relies on the use of volleys and smashing actions. Defensive actions are focused on sending the opponent to the back of the court though lobs and bouncing the ball off the wall from the baseline [5]. Thus, match activity in padel can be considered as a mix between tennis and squash [8,9].

Aside from game dynamics and technical-tactical demands, performance is also related with physical fitness and anthropometric measures. Particularly in racket sports, body kinematics and anthropometric parameters are associated with greater success [10,11]. Furthermore, recent research suggests that physical fitness has little impact on determining padel players’ performance at nonprofessional levels [12,13]. This may be due to the less demanding competitive requirements at a recreational level. Nonetheless, the increasing intensity of sport competitions at a professional level make strength and conditioning training a priority for success [14]. Although a few studies have examined anthropometric characteristics in amateur padel players [4,12,15], there is no information available to confirm these results in top level players. The determination of normative profiles for professional padel players would assist coaches in the talent identification process and improve the design of specific training in order to maximize performance.

The aims of the present study were (1) to describe and compare the anthropometric and physical fitness attributes of male padel players at different competitive levels, (2) to establish an anthropometric and physical profile chart for professional male padel players, and (3) to compare results of the present study with outcomes obtained in other racket sports.

## 2. Materials and Methods

### 2.1. Subjects

Sixty male padel players (27.7 ± 6.4 years) volunteered to participate in this study. They were participants at two Open events on the 2012 Padel Pro Tour (PPT) in Marbella and Fuengirola (Spain). PPT is the professional padel circuit, which was created between 2005 and 2013, and is currently called World Padel Tour (WPT). It constitutes the most important professional padel tournament in the world. Participants were classified according to competition level as (1) an elite group (n = 25) who competed in the Main Draw of PPT events (members of the top 28 pairings in the PPT ranking), and (2) a subelite group (n = 35) who competed in a Qualifying Round or in a Prequalifying Round for these events (players who were ranked below the top 28 pairings in the PPT ranking). Players were fully informed about the experimental procedures, including the risks and benefits of participation. Written informed consent was obtained from each player prior to data collection. The study was approved by the Ethics Committee of the University of Granada (number 883) and was carried out in compliance with the Declaration of Helsinki.

### 2.2. Anthropometric Data

Anthropometric measurements were performed according to guidelines outlined by the International Society for Advancement of Kinanthropometry (ISAK) [16]. All anthropometric variables were taken by the same experienced evaluator who was a Level 2 ISAK anthropometrist. Technical measurement error was less than 5% for skinfolds and less than 1% for all other measurements. Anthropometric variables included stature, body mass, arm span, 8 skinfolds (biceps, triceps, subscapular, suprailiac, supraspinal, abdominal, thigh, and medial calf), 7 girths (flexed and tensed upper arm, right and left forearms, wrist, thigh, and medial calf), 2 breadths (humeral and femoral), and width and length of the hands (dominant and nondominant). Stature was measured to the nearest 0.1 cm using a stadiometer (GPM, Seritex, Inc., Carlstadt, New Jersey), and body mass was measured to the nearest 0.1 kg using a portable scale (model 707, Seca Corporation, Columbia, Maryland). Skinfold thickness was obtained using a Holtain skinfold caliper (Holtain Ltd., Crymych, UK) and recorded to the nearest 0.2 mm. Girths were measured using a flexible anthropometric steel tape (Holtain Ltd., Crymych, UK) to the nearest 0.1 cm. Skinfolds were taken 3 times and the median was used in analyses. The sum of 3 skinfolds (triceps, subscapular, and supraspinal), 6 skinfolds (sum of 3 with suprailiac, abdominal, and thigh), and 8 skinfolds (sum of 6 with biceps and medial calf) was also calculated. Body mass index (BMI) was calculated as body mass/stature^2^ where body mass was expressed in kilograms (kg) and stature in metres (m). Five different equations [17,18,19,20,21] were used to estimate body density. Body fat percentage (BF%) was determined using Siri’s equation [22]. Muscle mass (MM) was determined in kg using the methods of Lee et al. [23]. Somatotype characteristics were determined according to the Carter and Heath method [24].

### 2.3. Lower-Body Muscular Strength

Following determination of anthropometric variables, players performed a warm-up consisting of several submaximal jumps. Lower-body muscular strength was assessed using the Infrared Platform Ergo Jump Plus-Bosco System (Byomedic, S.C.P., Barcelona, Spain). Each player completed three maximal countermovement jumps (CMJs) with 3 minutes rest between trials. The best score was recorded. All CMJs were completed keeping the hands on the hips throughout the test. Whilst standing erect, participants were instructed to flex their knees into a squat position (90) and then immediately rebound in a maximal vertical jump. No pause was allowed between the eccentric and concentric phase, and participants landed with both feet in contact with the floor. Measured height was expressed in centimetres and was converted to power (w) using the González-Badillo and Gorostiaga equation [25]:

$$Power\;\left(w\right)=weight\;\left(kg\right)\;\times 9.81\;\times \left[\sqrt{2\times 9.81\;\times jump\;height\;\left(m\right)}\;\right]$$

### 2.4. Handgrip Strength

A grip strength dynamometer (Takei Kiki Kogyo, Tokyo, Japan) was used to determine handgrip strength in both right and left hands. The dynamometer was adjusted for each participant’s hand size. Players maintained a standing position with the shoulder adducted and neutrally rotated and the elbow fully extended. The dynamometer was held freely without support, not touching the subject’s trunk. Players were instructed to perform a maximal isometric contraction for five seconds. Each participant completed three trials with each hand, with a 1 min rest between trials, and highest scores (in kg) were recorded.

### 2.5. Lumbar Isometric Strength

A lumbar extension dynamometer (Takei Kiki Kogyo, Tokyo, Japan) was used to determine lumbar isometric strength. Players stood on the platform with knees extended and the trunk flexed to an angle of 150°. Holding the bar with a pronated grip, the participant pulled it slowly, but vigorously, extending the lower back. Players were instructed to perform a maximal isometric contraction for five seconds. The best score of three trials with 1 min recovery between each was recorded and used in the analyses.

### 2.6. Flexibility

The sit-and-reach test was used to assess lower-body flexibility. A sit-and-reach box (Novel Products, Inc., Rockton, Illinois, USA) with a scale marked on the upper side was placed against a wall. Players removed their shoes and sat on the floor with their legs fully extended and feet against the box. Placing one hand on top of the other and keeping their legs straight, players reached forward as far as possible whilst sliding their fingers along the measurement scale on top of the box. Players were asked to hold the final position for three seconds, and measurements were recorded to the nearest centimetre. After a familiarization practice, each player performed three trials with the best score being recorded for analysis.

### 2.7. Statistical Analyses

Variables are described as mean, standard deviation, and range. The standardising of variables was carried out using the Shapiro–Wilk test. Differences in physical and anthropometric variables between the elite group and the subelite group of padel players were analysed using an independent *t*-test. Statistical significance was stablished at 5%. A profile chart with norms determined according to percentiles (values of 5, 10, 25, 50, 75, 90, and 95) was constructed for the elite group of male padel players. All statistical analyses were performed using the Statistical Package for Social Sciences (version 21.0; SPSS, Inc, Chicago, Illinois, USA).

## 3. Results

**Figure 1:**
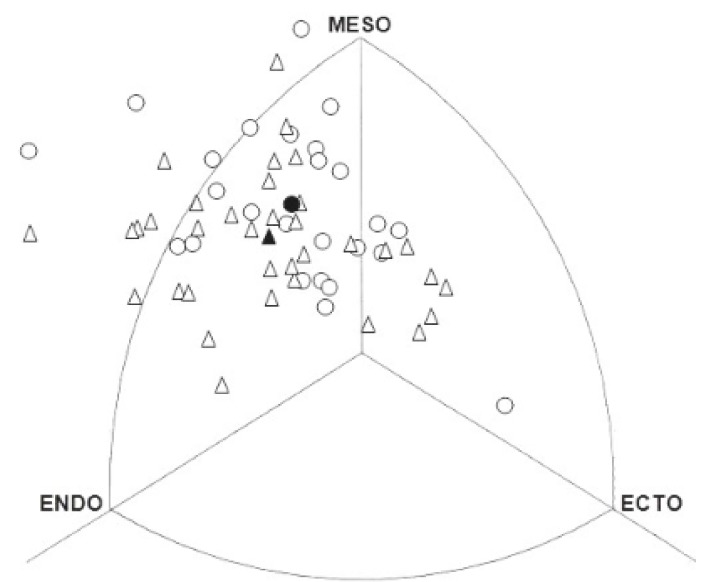
Somatotype distribution of elite (•: Mean somatotype = 3.7–5.7–2.0; ○: Individual somatotype) and subelite (▲: Mean somatotype = 4.3–5.3–2.1; ∆: Individual somatotype) padel players. MESO: mesomorphy; ECTO: ectomorphy; ENDO: endomorphy.

Demographic characteristics of the sample are presented in Table 1. Comparisons between groups showed that the elite padel players were significantly older (31 ± 5.7 vs. 25.3 ± 5.9 years), had significantly lower values for the thigh (10.6 ± 3.8 vs. 14.1 ± 5.7 mm) and calf (6.7 ± 2.3 vs. 9.3 ± 4.4 mm) skinfolds, the sum of six and eight skinfolds (84.5 ± 30.7 vs. 102.7 ± 38.5 mm and 95.3 ± 33.3 vs. 116 ± 43.2 mm, respectively), and the sum of lower-limb skinfolds (17.3 ± 5.4 vs. 23.5 ± 9.9 mm) than the subelite group. In the same way, elite padel players presented lower values for % body fat evaluated by Sloan [19], Wilmore and Behnke [20], and Whiters et al. [21] equations (9.7 ± 3.3 vs. 12.5 ± 4.7%, 14.2 ± 3.6 vs. 16.3 ± 4.2%, and 11.9 ± 4.1 vs. 14.6 ± 5.4%, respectively), and thigh fat area (27.5 ± 9.4 vs. 36.6 ± 15.4 cm^2^). Furthermore, elite players reported engaging in padel for longer (15.0 ± 6.1 vs. 8.6 ± 3.9 years) and showed significantly higher values for lumbar isometric strength (147.1 ± 43.5 vs. 126.9 ± 30.4 kg) than the subelite group. The mean somatotype for elite padel players was: 3.7–5.7–2.0. This demonstrates that these players were predominantly mesomorphic, being characterised as endo-mesomorphic according to Carter and Heath’s [24] classification. Figure 1 shows the somatoplots for all individual players.

Mean (± SD) hand grip strength of elite padel players overall was 51.3 ± 12.2 kg for the dominant hand and 43.6 ± 8.8 kg for the nondominant hand. Lumbar isometric strength, flexibility, and countermovement jump height were 147.1 ± 43.5 kg, 23.9 ± 9.6 cm, and 44.6 ± 5.3 cm, respectively. An anthropometric and physical profile is given in Table 2. Scores for 24 anthropometric dimensions and four performance tests are located on the chart, together with corresponding percentile values.

## 4. Discussion

The association between anthropometric characteristics and physical fitness in sports performance has been widely described in scientific literature [26,27,28]. However, this is the first study to our knowledge that has examined differences between male padel players according to their competitive level.

Comparisons between elite and subelite groups revealed elite players to be older (31 ± 5.7 vs. 25.3 ± 5.9 years) with a significantly greater number of training years (15.0 ± 6.1 vs. 8.6 ± 3.9 years). Furthermore, elite padel players showed lower values for thigh and calf skinfolds (10.6 ± 3.8 vs. 14.1 ± 5.7; 6.7 ± 2.3 vs. 9.3 ± 4.4, respectively), the sum of six and eight skinfolds (84.5 ± 30.7 vs. 102.7 ± 38.5 mm; 95.3 ± 33.3 vs. 116 ± 43.2 mm, respectively), and the sum of lower-limb skinfolds (17.3 ± 5.4 vs. 23.5 ± 9.9 mm), than the subelite padel players group. In addition, elite players showed lower BF% (between 2.1 and 2.7%) using three different equations [9,10,11], in addition to a lower thigh fat area (−9.1 cm^2^). This lower BF% could be related to the higher amount of training undertaken by the elite group. No statistically significant differences were observed between groups for height, body weight, and BMI. The mean somatotype for elite padel players (3.7–5.7–2.0) demonstrates that these athletes were predominantly mesomorphic, being characterised as endo-mesomorphic according to Carter and Heath [24].

Table 3 shows the anthropometric characteristics of different racket spots according to performance level and age.

With respect to padel players participating in a national university padel championship [5], players in our study were older (31.1 ± 5.7 years vs. 23.1 ± 3.6 years), heavier (77.2 ± 9.9 kg vs. 74.3 ± 8.6 kg), and shorter (177.7 ± 7.3 cm vs. 180.0 ± 10.0 cm). We also found lower values for the thickness of all skinfolds, higher thigh and calf girths (53.9 ± 3.2 cm vs. 52.2 ± 3.6 cm, 37.8 ± 2.3 kg vs. 37.1 ± 1.9 cm, respectively), higher mesomorphic component (5.7±1.2 vs. 4.1 ± 0.9), and similar endomorphic and mesomorphic components (3.7 ± 1.3 vs. 3.7 ± 1.2, 2.0 ± 1.1 vs. 2.4 ± 0.8, respectively). Similar results were observed by Castillo-Rodríguez et al. [36] in body weight, stature, and all components of somatotype. The only exception to this was found for the mesomorphic component, with this showing a lower value (5.7 ± 1.2 vs. 6.9 ± 1.3) than that reported for padel players in the present study.

We detected important differences between current elite padel players and athletes from other racket sports. Elite padel players were heavier (77.2 ± 9.9 kg vs. 71.7 ± 5.7 kg), with similar stature, higher skinfold thickness, and lower thigh girth than that observed in Spanish badminton players [29]. In addition, they showed higher endomorphic and mesomorphic components, and a lower ectomorphic component (3.7 ± 1.3 vs. 2.3 ± 0.6, 5.7 ± 1.2 vs. 3.7 ± 0.9, 2.0 ± 1.1 vs. 2.8 ± 0.9, respectively) than badminton players. Conversely, in comparison with previous reports in squash [44], current elite padel players were shorter (177.7 ± 7.3 cm vs. 180.0 ± 10.0 cm) and lighter (77.2 ± 9.9 kg vs. 78.7 ± 4.4 kg), showed higher values for skinfold thickness (biceps: 4.1 ± 1.5 mm vs. 3.8 ± 1.4 mm; triceps: 10.5 ± 4.4 mm vs. 6.7 ± 3.0 mm; subscapular: 10.1 ± 4.1 mm vs. 8.6 ± 2.1 mm; suprailiac: 18.3 ± 7.2 mm vs. 5.7 ± 2.2 mm; thigh: 10.6 ± 3.8 mm vs. 9.1 ± 4.3 mm; and calf: 6.7 ± 2.3 mm vs. 4.0 ± 2.5 mm), and a lower thigh girth (53.9 ± 3.2 cm vs. 58.5 ± 0.5 cm). Furthermore, mean somatotype of padel players in the present study was highly endomorphic and mesomorphic, but less ectomorphic (3.7 ± 1.3 vs. 2.5 ± 1.1, 5.7 ± 1.2 vs. 4.8 ± 0.5, 2.0 ± 1.1 vs. 2.9 ± 0.4, respectively). In comparison to table tennis players [29], elite padel players were taller and heavier (177.7 ± 7.3 cm vs. 174.2 ± 1.0 cm, 77.2 ± 9.9 kg vs. 69.7±1.2 kg, respectively), and showed a different somatotype with a greater endomorphic component (5.7 ± 1.2 vs. 3.9). Similarly, in comparison to subelite tennis players [6], the current padel players were taller and heavier (177.7 ± 7.3 cm vs. 180.0 ± 10.0 cm, 77.2 ± 9.9 kg vs. 74.7 ± 7.5 kg, respectively), showing similar somatotype components. The only exception in this case was seen in the mesomorphic component, with padel players showing higher values (5.7 ± 1.2 vs. 4.3 ± 1.2). Competition level may, however, account for these differences. Second- and third-division tennis players [57] were taller (184.8 ± 4.8 cm vs. 177.7 ± 7.3 cm), showed lower values for skinfold thickness (triceps: 7.3 ± 2.8 mm vs. 10.5 ± 4.4 mm; subscapular: 9.7 ± 1.9 mm vs. 10.1 ± 4.1 mm; suprailiac: 8.4 ± 2.7 mm vs. 18.3 ± 7.2 mm; thigh: 6.8±2.0 mm vs. 10.6 ± 3.8 mm; and calf: 7.8 ± 2.7 mm vs. 6.7 ± 2.3 mm), and a lower thigh girth (52.4 ± 3.0 cm vs. 53.9 ± 3.2 cm) than elite padel players. Nevertheless, elite padel players showed higher endomorphic and mesomorphic components and a lower ectomorphic component (3.7 ± 1.3 vs. 2.3 ± 0.6, 5.7 ± 1.2 vs. 3.2 ± 0.9, 2.0 ± 1.1 vs. 3.1 ± 0.8, respectively) than nonelite tennis players [11].

A player’s height and arm span is highly important in padel. High power is required to successfully bring rallies to a close, with the most powerful plays being found when players hit the ball when it is high above the head, as is often seen in tennis [5]. In this sense, it would be useful for padel players to be tall and of relatively high weight (higher muscle weight, of course) so that they are able to perform very powerful hits, especially when they start a point or when they want to finish it suddenly with a powerful shot (which can sometimes take the ball out of court). This situation does not normally happen in squash or in table-tennis. Padel players typically need to move their position more than squash or table tennis players, covering a bigger space. In addition, whilst they do not normally need such a fast reaction time, they do require high power in their legs to quickly shift their upper-body weight.

A main contribution of the current study was the measurement of physical fitness in world-class padel players. In contrast to what was observed in other racket sports like tennis or squash [9], no significant differences were found between elite and subelite groups in analysed fitness variables (CMJ, handgrip, and flexibility). The only exception was seen with lumbar isometric strength, which was greater in elite padel players (147.1 ± 43.5 kg vs. 126.9 ± 30.4 kg). Similar physical fitness between high- and low-level players has been previously observed in nonprofessional padel samples [12,13]. This appears to indicate that technical, tactical, and psychological aspects should become topics of interest in padel performance training and assessment. For instance, smashing abilities from middle distances (a low-frequency action <10% of the game, but executed at high speed) and volleying effectively close to the net (a high-frequency action >30% of the game and made in a short time frame) appear to be relevant for increasing point scoring [5]. In this sense, a stronger core musculature seems to be important in padel to allow players to perform powerful strokes. According to our results, better lumbar isometric strength could help padel players avoid lower back pain and develop greater hitting force when playing overhead groundstrokes involving ballistic trunk movements, such as a smash. This has also been reported in tennis [58].

## 5. Limitations

Results must be considered as a reference point, as opposed to being used as an obligatory model of better performance. In this way, the results presented can be used as a standard reference, but should be interpreted with caution according to individual characteristics and needs.

Strengths: The main strengths of the present study are that a) it is the first time that anthropometrical, somatotype, and body composition variables were measured in top-level padel players; b) participants were involved in performing padel at a high level (they were the best padel players in the world at the time of study); c) the best padel players were compared with lower ranked players in order to ascertain whether differences could be found with regards to anthropometric variables; d) the study was carried out during one of the most important competitions in the world for elite padel players and so all participants should have been in top physical shape. 

Future lines: a) To conduct a longitudinal study to investigate anthropometry, body composition, and somatotype of padel players at different competitive levels; b) to investigate nutritional habits and profiles in elite padel players, and their relationship with body composition and sports training.

Practical applications: This study provides reference values in relation to the anthropometric and physical characteristics of elite padel players. This information provides a frame of reference for coaches to control the training process in order to improve athletes’ performance and to facilitate talent detection and identification in padel.

## 6. Conclusions

In summary, the present study examined differences in the anthropometric characteristics and physical performance of padel players according to their competitive level (elite and subelite). Elite players showed lower values than subelite padel players for thigh and calf skinfolds, the sum of 6 and 8 skinfolds, and the sum of lower-limb skinfolds. Elite players also showed lower BF%. This study provides normative data that could help coaches with talent detection. Result may also help coaches in the design of training programs in order to achieve maximum sports performance.

## Figures and Tables

**Table 1:** Anthropometric and physical characteristics of the total sample, and elite and subelite padel players.

Variables	Total Padel Players (n = 60)		Elite (n = 25)		Subelite (n = 35)		*p*-Value	Cohen’s *d*
	Mean ± SD	Range	Mean ± SD	Range	Mean ± SD	Range		
Age (yr)	27.7 ± 6.4	15.2–40.2	31.1 ± 5.7	18.1–38.3	25.3 ± 5.9	15.2–40.2	0.000 **	0.505
Total years playing padel (yr)	12.5 ± 6.1	4.0–22.0	15.0 ± 6.1	8.0–22.0	8.6 ± 3.9	4.0–16.0	0.010 *	1.250
Training (hours/week)	9.2 ± 7.3	2.0–25.0	11.4 ± 7.8	2.0–25.0	5.8 ± 5.2	2.0–18.0	0.091	0.844
Stature (cm)	178.0 ± 7.5	163.5–200.2	177.7 ± 7.3	163.5–196.2	178.3 ± 7.7	164.1–200.2	0.772	−0.080
Body weight (kg)	77.5 ± 10.0	56.0–101.6	77.2 ± 9.9	56.0–97.9	77.6 ± 10.2	56.8–101.6	0.879	−0.040
BMI (kg/m^2^)	24.4 ± 2.3	18.5–31.4	24.4 ± 2.5	18.5–31.4	24.4 ± 2.3	19.0–28.7	0.933	0
Arm span (cm)	182.3 ± 8.3	161.5–203.5	182.3 ± 7.6	161.5–203.5	182.3 ± 8.9	164.5–201.5	0.990	0
Triceps skinfold (mm)	11.5 ± 4.9	5.2–26.8	10.5 ± 4.4	5.4–22.6	12.1 ± 5.2	5.2–26.8	0.204	−0.332
Biceps skinfold (mm)	4.6 ± 1.9	2.0–11.0	4.1 ± 1.5	2.4–8.2	4.9 ± 2.1	2.6–11.2	0.120	−0.438
Subscapular skinfold (mm)	10.9 ± 4.2	6.2–27.0	10.1 ± 4.1	6.4–27.0	11.5 ± 4.2	6.2–23.2	0.197	−0.337
Suprailiac skinfold (mm)	20.6 ± 8.7	8.2–39.2	18.3 ± 7.2	9.4–34.2	22.2 ± 9.3	8.2–39.2	0.083	−0.469
Supraspinale skinfold (mm)	9.9 ± 5.3	4.0–31.2	8.8 ± 4.2	4.2–22.2	10.7 ± 5.9	4.0–31.2	0.168	−0.371
Abdominal skinfold (mm)	18.9 ± 9.4	5.6–37.0	16.8 ± 8.7	6.2–36.2	20.5 ± 9.7	5.6–37.0	0.131	−0.402
Thigh skinfold (mm)	12.7 ± 5.3	5.8–31.6	10.6 ± 3.8	5.8–22.4	14.1 ± 5.7	6.6–31.6	0.009 *	−0.723
Calf skinfold (mm)	8.3 ± 3.9	4.0–22.0	6.7 ± 2.3	4.2–11.8	9.3 ± 4.4	4.0–22.0	0.004 *	−0.741
Upper-arm girth (cm)^†^	31.5 ± 2.5	25.8–37.1	31.8 ± 2.6	26.3–37.1	31.3 ± 2.4	25.8–36.3	0.434	0.200
Upper-arm girth (cm)^††^	33.6 ± 2.6	28.5–29.7	33.9 ± 2.6	28.2–38.0	33.4 ± 2.6	27.8–39.8	0.535	0.192
Right forearm girth (cm)	28.7 ± 1.8	24.6–33.2	28.8 ± 2.1	25.8–33.2	28.6 ± 1.7	24.6–31.5	0.659	0.105
Left forearm girth (cm)	27.5 ± 1.6	23.3–31.1	27.5 ± 1.8	23.3–31.1	27.4 ± 1.4	23.9–29.8	0.804	0.062
Wrist girth (cm)	17.4 ± 1.0	15.3–20.0	17.5 ± 1.1	15.6–20.0	17.2 ± 0.8	15.3–19.0	0.200	0.312
Thigh girth (cm)	53.8 ± 3.4	46.2–61.7	53.9 ± 3.2	47.2–61.7	53.7 ± 3.6	46.2–60.4	0.821	0.059
Calf girth (maximum) (cm)	37.8 ± 2.1	32.6–42.1	37.8 ± 2.3	32.6–41.8	37.8 ± 1.9	33.1–42.1	0.975	0
Humerus breadth (cm)	7.2 ± 0.4	6.2–8.4	7.3 ± 0.5	6.2–8.4	7.1 ± 0.4	6.4–8.0	0.240	0.442
Femur breadth (cm)	10.1 ± 0.5	8.9–11.3	10.1 ± 0.5	9.3–11.3	10.0 ± 0.5	8.9–11.0	0.395	0.200
Dominant hand length (cm)	20.0 ± 1.1	17.7–22.8	20.0 ± 1.1	17.7–22.2	20.0 ± 1.1	18.5–22.8	0.846	0
Dominant hand width (cm)	22.4 ± 1.7	18.9–26.5	22.3 ± 1.9	19.5–25.9	22.5 ± 1.5	18.9–26.5	0.746	−0.117
Nondominant hand length (cm)	19.8 ± 1.2	16.6–23.5	19.8 ± 1.3	16.6–22.8	19.9 ± 1.1	18.3–23.5	0.728	−0.083
Nondominant hand width (cm)	22.7 ± 1.6	19.3–26.7	22.6 ± 1.9	19.7–25.9	22.9 ± 1.4	19.3–26.7	0.563	−0.180
Sum of 3 skinfolds (mm)	42.9 ± 16.6	19.6–88.0	38.9 ± 14.6	22.2–83.8	45.8 ± 17.5	19.6–88.0	0.109	−0.428
Sum of 6 skinfolds (mm)	95.1 ± 36.4	40.0–186.0	84.5 ± 30.7	49.0–164.6	102.7 ± 38.5	40.0–186.2	0.047 *	−0.523
Sum of 8 skinfolds (mm)	107.9 ± 40.5	49.0–210.0	95.3 ± 33.3	58.0–184.4	116.9 ± 43.2	48.6–210.4	0.041 *	−0.560
Sum upper-limb skinfolds (mm)	76.3 ± 31.6	32.2–150.4	68.5 ± 27.8	36.6–150.4	81.9 ± 33.3	32.2–150.2	0.105	−0.437
Sum lower-limb skinfolds (mm)	20.9 ± 8.8	10.2–53.0	17.3 ± 5.4	10.2–34.2	23.5 ± 9.9	11.6–53.0	0.003 *	−0.798
% body fat								
Durnin and Womersley [17]	19.7 ± 5.3	10.1–31.2	18.4 ± 4.8	11.7–30.4	20.7 ± 5.4	10.1–31.2	0.106	−0.450
Katch and McArdle [18]	13.4 ± 5.1	6.3–27.4	12.2 ± 4.7	7.2–26.7	14.2 ± 5.3	6.3–27.4	0.139	−0.399
Sloan [19]	11.3 ± 4.4	5.2–24.0	9.7 ± 3.3	5.7–19.2	12.5 ± 4.7	5.2–24.0	0.012 *	−1.182
Wilmore and Behnke [20]	15.4 ± 4.1	9.3–24.3	14.2 ± 3.6	9.5–21.8	16.3 ± 4.2	9.3–24.3	0.050 *	−0.537
Withers et al. [21]	13.5 ± 5.1	6.5–26.2	11.9 ± 4.1	7.0–24.4	14.6 ± 5.4	6.5–26.2	0.037 *	−0.563
Skeletal muscle mass (kg), Lee et al. [23]	33.1 ± 3.9	25.4–44.1	33.9 ± 4.2	26.2–44.1	32.5 ± 3.7	25.4–41.4	0.181	0.354
Total upper-arm area (cm^2^)	79.6 ± 12.5	53.0–109.5	81.1 ± 13.1	55.0–109.5	78.5 ± 12.1	53.0–104.9	0.422	0.206
Upper-arm muscle area (cm^2^)	67.4 ± 10.7	47.8–94.1	69.9 ± 11.4	49.2–90.3	65.6 ± 10.0	47.8–94.1	0.132	0.401
Upper-arm fat area (cm^2^)	12.2 ± 5.3	5.2–31.6	11.3 ± 4.7	5.9–26.7	12.9 ± 5.7	5.2–31.6	0.257	−0.306
Total thigh area (cm^2^)	231.2 ± 28.9	169.9–302.9	232.1 ± 27.4	177.3–302.9	230.5 ± 30.3	169.9–290.3	0.844	0.055
Thigh muscle area (cm^2^)	198.4 ± 26.5	142.8–274.6	204.5 ± 27.9	154.0–274.6	194.0 ± 25.0	142.8–247.3	0.131	0.396
Thig fat area (cm^2^)	32.8 ± 13.9	14.8–82.7	27.5 ± 9.4	14.8–55.9	36.6 ± 15.4	15.5–82.7	0.007 *	−0.713
Somatotype								
Endomorphy	4.1 ± 1.5	1.8–7.9	3.7 ± 1.3	2.0–7.3	4.3 ± 1.5	1.8–7.9	0.126	−0.427
Mesomorphy	5.4 ± 1.1	2.6–8.0	5.7 ± 1.2	2.7–8.0	5.3 ± 0.9	3.5–7.3	0.138	0.377
Ectomorphy	2.1 ± 1.1	−0.6 to 5.4	2.0 ± 1.1	−0.6 to 5.4	2.1 ± 1.1	0.0–4.3	0.879	−0.090
Hand grip strength								
Dominant hand (kg)	49.4 ± 9.7	36.3–85.2	51.3 ± 12.5	36.6–85.2	48.1 ± 7.1	36.3–69.9	0.268	0.315
Nondominant hand (kg)	42.7 ± 6.6	25.7–63.8	43.6 ± 8.2	25.7–63.8	42.1 ± 5.3	31.0–52.7	0.407	0.217
Sum two hands (kg)	92.1 ± 14.8	65.1–149.0	94.9 ± 18.8	65.1–149.0	90.2 ± 11.2	67.3–122.6	0.239	0.304
Lumbar isometric strength (kg)	135.2 ± 37.4	28.0–275.0	147.1 ± 43.5	68.0–275.0	126.9 ± 30.4	28.0–186.0	0.042 *	0.538
Flexibility (cm)	22.1 ± 8.6	4.0–41.0	23.9 ± 9.6	4.0–41.0	20.8 ± 7.6	4.0–35.0	0.175	0.358
Lower-body muscular strength								
CMJ height (cm)	43.3 ± 5.9	31.0–59.0	44.6 ± 5.3	34.0–54.0	42.3 ± 6.2	31.0–59.0	0.159	0.399
CMJ power (W)	2212.5 ± 352.8	1500.0–3311.0	2257.0 ± 336.2	1500.0–3126.1	2179.1 ± 366.4	1541.3–3311.0	0.418	0.222

* *p* < 0.05; ** *p* < 0.001; † Relaxed; †† Tensed. CMJ: Countermovement jump. BMI: Body mass index.

**Table 2:** Percentiles for the anthropometric and physical variables of elite padel players (n = 25).

Variable	Percentiles						
	5	10	25	50	75	90	95
Height (cm)	164.4	166.9	174.3	177.6	182.8	187.1	194.5
Weight (kg)	58.1	64.9	71.3	77.1	81.2	94.2	97.5
BMI (kg·m^−2^)	19.2	21.6	23.1	24.3	25.6	27.5	30.3
Arm span (cm)	165.4	175.6	178.8	181.8	184.7	192.8	201.0
Triceps skinfold (mm)	5.6	6.1	7.4	8.4	13.2	17.1	21.0
Biceps skinfold (mm)	2.4	2.5	2.9	4.0	4.9	6.7	7.8
Subscapular skinfold (mm)	6.5	7.0	7.6	9.4	11.2	13.0	22.9
Suprailiac skinfold (mm)	9.6	10.3	12.6	16.6	22.4	30.3	33.4
Supraespinale skinfold (mm)	4.3	4.7	5.6	7.4	11.1	14.6	20.2
Abdominal skinfold (mm)	6.4	7.1	9.7	13.2	23.4	30.7	35.1
Thigh skinfold (mm)	5.9	6.2	8.2	9.6	12.4	15.6	20.9
Calf skinfold (mm)	4.2	4.3	5.0	5.8	8.6	10.3	11.7
Upper-arm girth (cm) †	26.8	28.2	30.1	31.5	33.6	35.2	36.6
Upper-arm girth (cm) ††	28.6	29.9	31.9	34.2	35.4	37.7	38.0
Right forearm girth (cm)	25.9	26.2	27.1	28.6	30.3	32.3	33.1
Left forearm girth (cm)	23.7	24.9	26.6	27.5	28.7	30.3	31.0
Wrist girth (cm)	15.7	16.1	16.7	17.6	18.3	19.2	19.9
Thigh girth (cm)	47.9	50.2	51.3	53.7	56.0	58.0	60.8
Calf girth (maximum) (cm)	33.1	34.7	36.4	38.1	39.5	41.2	41.6
Humerus breath (cm)	6.3	6.6	6.9	7.4	7.6	8.1	8.3
Femur breath (cm)	9.3	9.4	9.7	10.1	10.5	10.8	11.2
Dominant hand length (cm)	17.8	18.5	19.4	20.0	20.6	21.8	22.1
Dominant hand width (cm)	19.5	19.9	20.6	22.5	23.8	25.0	25.8
Nondominant hand length (cm)	16.8	18.0	19.3	19.7	20.4	21.4	22.5
Nondominant hand width (cm)	19.7	19.9	20.9	22.5	24.7	25.0	25.7
Hand grip strength							
Dominant hand (kg)	37.3	39.5	42.9	46.5	57.8	74.1	84.1
Nondominant hand (kg)	27.7	33.6	38.7	43.1	47.5	55.9	62.7
Sum of two hands (kg)	66.4	73.2	83.1	91.3	102.9	126.2	143.5
Lumbar isometric strength (kg)	75.1	101.8	120.8	143.5	160.1	216.8	263.5
Flexibility (cm)	4.5	9.5	19.0	24.0	30.8	39.0	40.8
Lower-body muscular strength							
CMJ Height (cm)	34.3	36.5	40.8	45.0	47.8	52.0	54.0
CMJ Power (W)	1606.5	1959.1	2032.6	2213.9	2405.6	2788.1	3079.0

† Relaxed; †† Flexed and tensed; CMJ: Countermovement jump.

**Table 3:** Summary table of studies examining age, stature, body weight, body mass index (BMI), body fat percentage and somatotype of elite players of different racket sports (mean ± SD).

Study	n	Age (yr)	Stature (cm)	Body Weight (kg)	BMI (kg·m^−2^)	Body Fat (%)	Endomorphy	Mesomorphy	Ectomorphy	Event
Abián et al. [29]	31	21.7 ± 4.3	177.9 ± 6.0	71.7 ± 5.7	^#^	8.4 ± 1.4	2.3 ± 0.6	3.7 ± 0.9	2.8 ± 0.9	Badminton
Amusa et al. [30]	6	17.5 ± 0.5	171.0 ± 4.7	54.8 ± 3.0	18.7 ± 0.8	6.9 ± 1.7	^#^	^#^	^#^	Badminton
Berral de la Rosa et al. [31]	37	17.2 ± 0.9	169.7 ± 7.7	63.9 ± 7.1	^#^	13.3 ± 2.2 13.0 ± 2.4	^#^	^#^	^#^	Badminton
Buti et al. [32]	8	11.7	147.9 ± 2.9	38.5 ± 4.8	^#^	^#^	^#^	^#^	^#^	Tennis
Campos et al. [33]	10	17.2 ± 1.3	172.4 ± 1.0	68.0 ± 7.8	22.4 ± 2.0	^#^	^#^	^#^	^#^	Badminton
Canaki et al. [34]	24	16	178.4 ± 8.4	65.6 ± 8.2	20.5 ± 1.4	8.5 ± 2.9	2.8 ± 0.9	3.6 ± 0.9	3.7 ± 0.8	Tennis
	25	18	184.2 ± 6.9	75.5 ± 8.3	22.2 ± 1.9	8.7 ± 3.8	2.9 ± 0.8	3.3 ± 0.9	3.4 ± 1.0	Tennis
Carrasco et al. [35]	38	11.3 ± 1.8	149.1 ± 12.2	41.6 ± 1.8	18.4 ± 2.5	^#^	3.6 ± 1.4	4.6 ± 0.7	3.3 ± 1.2	Table tennis
Castillo-Rodríguez et al. [36]	36	27.0 ± 5.2	177.4 ± 6.4	78.5 ± 8.7	24.9 ± 2.5	^#^	3.6 ± 1.1	6.9 ± 1.3	1.9 ± 1.0	Padel
Centeno Prada et al. [37]	11	15.6 ± 1.4	177.3 ± 5.1	67.2 ± 5.2	^#^	11.7 ± 1.6	2.6 ± 0.5	4.1 ± 0.5	3.4 ± 0.8	Badminton
Chin et al. [38]	10	20.7 ± 2.5	172.6 ± 4.3	67.7 ± 6.9	^#^	7.4 ± 3.4	^#^	^#^	^#^	Squash
Eiben and Eiben [39]	48	23.5	174.2 ± 1.0	69.7 ± 1.2	^#^	^#^	3.5	3.9	2.5	Table tennis
Elliott et al. [40]	17	11	142.4 ± 5.7	33.5 ± 4.0	^#^	^#^	2.2 ± 0.8	4.1 ± 0.7	3.9 ± 1.0	Tennis
	27	13	153.4 ± 7.7	41.1 ± 6.3	^#^	^#^	2.2 ± 1.0	3.9 ± 0.7	4.1 ± 1.0	Tennis
	13	15	169.6±8.3	54.0 ± 8.8	^#^	^#^	1.9 ± 0.6	3.9 ± 1.0	4.5 ± 1.1	Tennis
Gahlot [41]	30	18–25	175.0±2.0	69.8 ± 6.7	24.2 ± 2.0	13.2 ± 3.4	^#^	^#^	^#^	Badminton
Heller [42]	29	17.2 ± 1.2	183.2±5.5	71.2 ± 7.4	21.2 ± 2.1	6.1 ± 2.6	^#^	^#^	^#^	Badminton
	25	21.3 ± 2.2	182.0±4.3	75.1 ± 3.6	22.7 ± 1.2	8.3 ± 2.6	^#^	^#^	^#^	Badminton
HuanYu et al. [43]	12	16.7 ± 1.6	171.8±5.4	60.5 ± 1.9	^#^	^#^	^#^	^#^	^#^	Table tennis
Jaski and Bale [44]	6	27.3 ± 3.6	180.2±7.7	78.7 ± 4.4	^#^	10.2 ± 3.3	2.5±1.1	4.8 ± 0.5	2.9 ± 0.4	Squash
Majumdar et al. [45]	6	24.3 ± 4.1	175.4±5.4	64.8 ± 6.9	^#^	12.1 ± 3.4	^#^	^#^	^#^	Badminton
Mathur et al. [46]	18	22.3 ± 2.4	172.4±5.3	67.9 ± 3.6	^#^	8.2 ± 1.7	2.2 ± 0.9	3.9 ± 1.1	2.9 ± 0.6	Badminton
Munivrana et al. [47]	62	12.8 ± 1.7	^#^	^#^	^#^	^#^	2.8 ± 1.2	3.7 ± 0.9	3.5 ± 1.3	Table tennis
Poliszczuk and Mosakowska [48]	9	22.3 ± 2.4	184.6±6.0	80.7 ± 9.1	23.6 ± 2.0	^#^	3.0	3.0	2.5	Badminton
Pradas de la Fuente et al. [49]	38	11.3 ± 1.8	149.1 ± 12.2	41.6 ± 1.8	18.4 ± 2.5	13.6 ± 4.6	3.6 ± 1.4	4.6 ± 0.7	3.3 ± 1.2	Table tennis
Pyke et al. [50]	15	25.6	177.1 ± 5.9	76.8 ± 9.0	^#^	12.0 ± 2.0	^#^	^#^	^#^	Squash
	5	16.2	174.7 ± 1.4	64.3 ± 6.5	^#^	10.4 ± 1.2	^#^	^#^	^#^	Tennis
Revan et al. [51]	12	16.4 ± 0.7	175.0 ± 7.3	67.5 ± 7.7	22.0 ± 2.0	10.9 ± 2.1	2.0 ± 0.4	3.6 ± 1.5	3.0 ± 1.0	Badminton
Sánchez-Muñoz et al. [52]	57	16.2 ± 0.4	176.8 ± 6.4	69.9 ± 6.8	22.3 ± 1.4	15.8 ± 3.6	2.4 ± 0.7	5.2 ± 0.8	2.9 ± 0.7	Tennis
Ulbricht et al. [53]	54	15.0–15.5	177.4 ± 6.5	65.5 ± 7.3	20.8 ± 1.7	^#^	^#^	^#^	^#^	Tennis
Ulbricht et al. [54]	24	11.5 ± 0.3	151.2 ± 7.0	40.5 ± 5.6	17.6 ± 1.4	^#^	^#^	^#^	^#^	Tennis
	26	13.1 ± 0.5	165.2 ± 8.6	49.1 ± 8.1	17.9 ± 1.5	^#^	^#^	^#^	^#^	Tennis
	28	15.0 ± 0.5	179.1 ± 6.3	65.3 ± 7.4	20.3 ± 1.5	^#^	^#^	^#^	^#^	Tennis
Wan Nudri et al. [55]	7	16.4 ± 0.9	167.0 ± 5.0	61.3 ± 4.9	22.0 ± 1.8	14.6 ± 2.9	^#^	^#^	^#^	Badminton
Yasin et al. [56]	15	21.7 ± 3.5	-	72.0 ± 6.0	^#^	^#^	^#^	^#^	^#^	Badminton
	15	21.1 ± 3.5	-	74.6 ± 3.5	^#^	^#^	^#^	^#^	^#^	Tennis
Present study	25	31.1 ± 5.7	177.7 ± 7.3	77.2 ± 9.9	24.4 ± 2.5	18.4 ± 4.8 12.2 ± 4.7 9.7 ± 3.3 14.2 ± 3.6 11.9 ± 4.1	3.7 ± 1.3	5.7 ± 1.2	2.0 ± 1.1	Padel

^#^ Data not availaible.
